# Slimy partners: the mucus barrier and gut microbiome in ulcerative colitis

**DOI:** 10.1038/s12276-021-00617-8

**Published:** 2021-05-17

**Authors:** Jian Fang, Hui Wang, Yuping Zhou, Hui Zhang, Huiting Zhou, Xiaohong Zhang

**Affiliations:** 1 Department of Preventive Medicine, Zhejiang Key Laboratory of Pathophysiology, School of Medicine, Ningbo University, 818 Fenghua Road, Ningbo, Zhejiang People’s Republic of China; 2 College of Medicine, Shaoxing University, 508 Huancheng Road, Shaoxing, Zhejiang Province People’s Republic of China; 3 Department of Colorectal Surgery, Shaoxing people’s Hospital, 568 North Zhongxing Road, Shaoxing, Zhejiang Province People’s Republic of China; 4 The Affiliated Hospital of Medical School, Ningbo University, 247 Renmin Road, Ningbo, Zhejiang People’s Republic of China

**Keywords:** Glycobiology, Ulcerative colitis

## Abstract

Ulcerative colitis (UC) is a chronic recurrent intestinal inflammatory disease characterized by high incidence and young onset age. Recently, there have been some interesting findings in the pathogenesis of UC. The mucus barrier, which is composed of a mucin complex rich in O-glycosylation, not only provides nutrients and habitat for intestinal microbes but also orchestrates the taming of germs. In turn, the gut microbiota modulates the production and secretion of mucins and stratification of the mucus layers. Active bidirectional communication between the microbiota and its ‘slimy’ partner, the mucus barrier, seems to be a continually performed concerto, maintaining homeostasis of the gut ecological microenvironment. Any abnormalities may induce a disorder in the gut community, thereby causing inflammatory damage. Our review mainly focuses on the complicated communication between the mucus barrier and gut microbiome to explore a promising new avenue for UC therapy.

## Gut microbiome: Slimy symbiosis

Gaining a better understanding of the bidirectional communication between the gut microbiome and the colon’s mucus layer, which covers and protects intestinal tissue, could aid in developing effective treatments for ulcerative colitis (UC). Increasing evidence links gut microbiome imbalance to UC, a form of inflammatory bowel disease. Xiaohong Zhang at Ningbo University in China and co-workers have reviewed gut microbiome–mucus layer interactions. They report that the mucus layer, comprised of a firm sterile inner layer, and a loose, densely colonized outer layer, nourishes and provides a home for ‘good’ microbes, while excluding pathogens. In turn, good microbes promote mucus layer production and stratification. Without adequate dietary fiber, gut microbes over-consume the mucus layer, exposing intestinal tissue and causing inflammation. This article provides clue for investigating treatments for inflammatory bowel diseases, including UC.

## Introduction

**Fig. 1:**
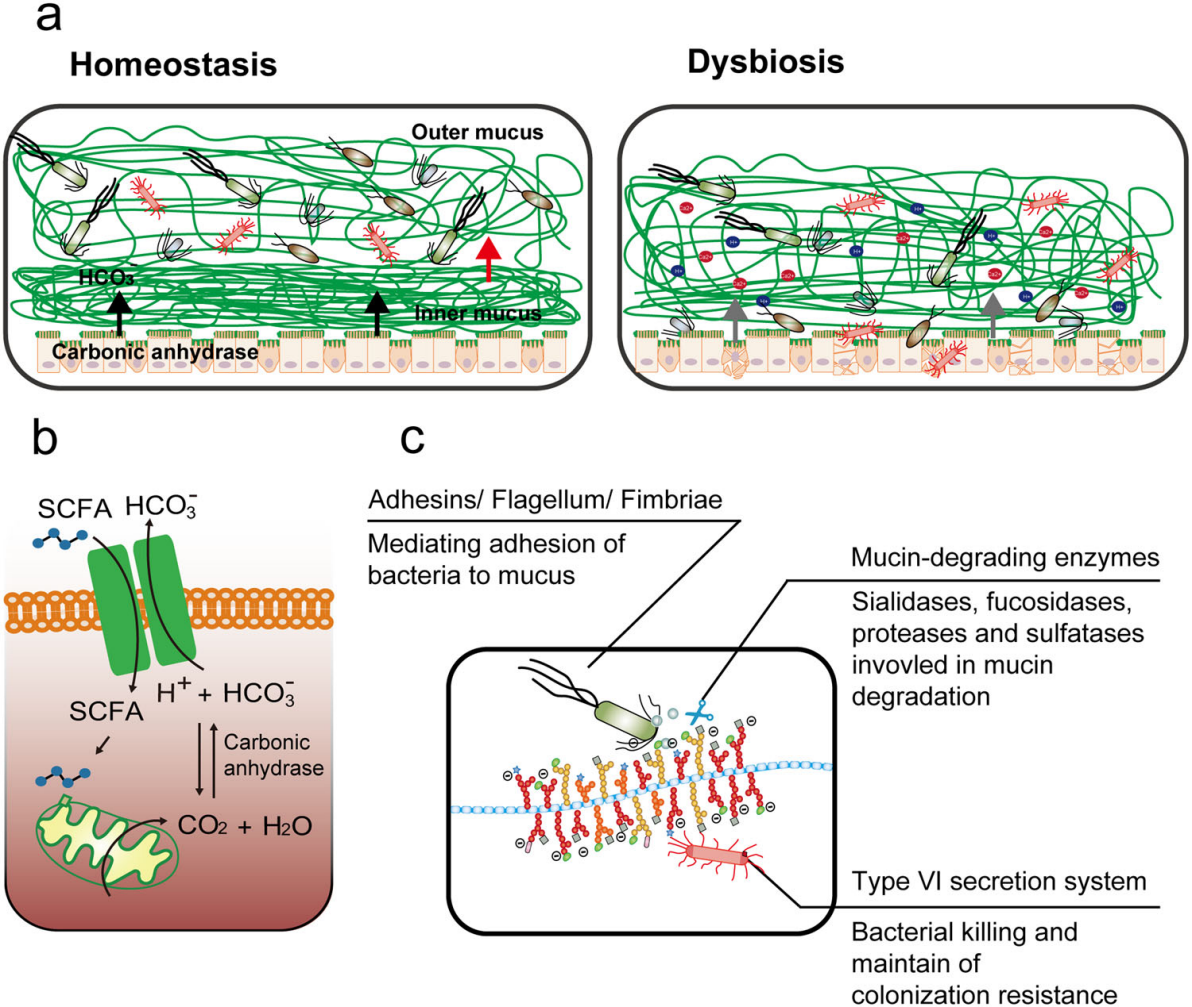
The gut microbiome acts as orchestrator of the mucus barrier. **a** During homeostasis, the gut microbiome at the outer mucus layer modulates mucin production and secretion and mucus stratification mediated by HCO_3_^−^ to maintain mucus barrier integrity. Dysbiosis induces impairment of the mucus barrier, accompanied by increased epithelium damage, bacterial translocation, goblet cell depletion, and host inflammation. **b** Gut microbiome-generated short-chain fatty acids enter colonocytes and are oxidized to generate CO_2_ that can be converted by carbonic anhydrase into HCO_3_^−^, which is the ideal physiological solution for precipitating calcium and raising the pH at the epithelial surface. This in turn promotes the stratification of the mucus layer. **c** Intestinal bacteria have evolved several strategies to adhere to the mucus barrier, including the use of adhesins, flagella, and fimbriae; achieve cross-feeding by mucin degradation; and maintain colonization resistance by means of a commensal type VI secretion system.

In recent years, the incidence of ulcerative colitis (UC), an inflammatory bowel disease (IBD) of unknown etiology, has been increasing globally, especially in some newly industrialized countries, including India and China^1^. Microbial infections such as those by *Clostridium difficile* have been described as a mono-associated cause of UC flare-ups^2^; however, there is growing evidence that UC is an overly robust mucosal immune response to dysbiosis of particular gut flora that is characterized by abnormal microbiota composition and bacterial products^3,4^. A balanced microbiome community is vital for maintaining mucus barrier homeostasis, which involves a dynamic balance of production, secretion, expansion, and proteolysis of mucus components. Commensal bacteria and their fermentation products (short-chain fatty acids, SCFAs) are implicated in the regulation of the production and secretion of mucin 2 (Muc2), the major component of mucus, in sentinel goblet cells (sGCs) at crypt opening^5^ (Fig. 1a). The gut microbiome also influences the mucosal structure. Carbon dioxide (CO_2_) generated from β-oxidation of SCFAs in colonocytes is converted by carbonic anhydrase to bicarbonate (HCO_3_^−^), which in turn dictates the stratification of the mucus layers, such as the unfolding of mucin and resultant inner-towards-outer conversion of the mucus layer^6^ (Fig. 1b).

While the secreted, attached, hydrated, and stratified mucus barrier is mostly considered a simple lubricant layer overlying the epithelium, it also provides an environment for bacterial colonization and nourishes the commensal microbiota, thereby stabilizing the microbial community and promoting symbiotic interactions, resulting in microbial commensalism^7^. Mucus barrier abnormalities, including depleted upper crypt GCs, bacterial penetration of the inner mucus layer, and decreased core mucus components, such as FCGBP (human IgGFc binding protein), CLCA1 (calcium-activated chloride channel regulator 1), and ZG16 (zymogen granule protein 16), in active UC support the notion that an impaired mucus barrier may occur prior to the onset of inflammation in the pathogenesis of UC^8^. Environmental factors such as diet and lifestyle factors may shape the human gut microbiome composition, thereby influencing mucus homeostasis and the development of intestinal inflammatory lesions^9^. Dietary fiber-deprived intestinal microbiota consume components of the mucus layer, leading to intestinal barrier dysfunction and increased susceptibility to pathogens and colitis occurrence^10^. It is obvious that the interplay between the microbiota and its ‘slimy’ partner, the mucus barrier, in the gut is constitutive. Therefore, any attempt to simply explore the underlying mechanism of UC from any single part of the biosystem (the mucus barrier and gut microbiota) is unwise. Currently, the development of microbiome-targeted therapeutic strategies for mild to moderate UC is growing^11^, and mucus barrier-associated colonization resistance involves commensal bacteria out-competing foreign microbes for space, trophic resources and bactericidal factors in the mucus barrier and decreasing the efficacy of fecal microbiome transplantation (FMT) therapy. This review provides insight into mucus barrier-gut microbiome interactions.

## The gut microbiome: orchestrator of the mucus barrier

### The gut microbiome adheres to mucus

Compared with the small intestine, the colonic epithelium is covered by mucus layers composed of a firm inner layer and loose outer layer that function to separate microbes from epithelial cells and provide a diffusion barrier to maintain a balanced community. The outer mucus layer is colonized with an abundance of commensal microbes, while the inner layer is relatively sterile (Fig. 1a). The combination of the mucus barrier and gut microbiome, composed of approximately 100 trillion symbiotic microbial cells and more than 9000 carbohydrate-degrading enzymes, is described as “the last human body organ”^12^. Commensal bacteria and pathogens have evolved several strategies to occupy a narrowly defined niche within the mucus barrier.

The first strategy is to adhere to the mucus by surface display of adhesins and extracellular appendages (fimbria) that bind to specific mucin glycans (Fig. 1c). Mucus-binding proteins (MUBs) are one class of effectors involved in the adherence of lactobacilli, abundant commensal bacteria in the human gut and the best studied example of mucus adhesins that confine commensal/probiotic bacteria to the outer mucus layer^13^. Phylogenetically, adhesins are proteins characterized by the MUB domain, which shares homology with the Pfam-MucBP (mucin-binding protein) domains^14^. MUB and MucBP domain-containing proteins contain a C-terminal recognition motif (LPxTG) that is recognized by a family of enzymes called sortases for covalent attachment to peptidoglycan of the bacterial cell wall and an N-terminal region for protein secretion, in addition to a signal peptide (Table 1). A number of proteins containing MUB homologs and MucBP domains have been found; for instance, the mucin/mucin-binding protein of *Lactobacillus fermentum* BCS87 (32-Mmubp), S-layer protein in *L. acidophilus* (SlpA), MucBP-containing mannose-specific adhesin (Msa), and elongation factor Tu (EF-Tu) are highly prevalent in lactobacilli naturally existing in intestinal niches. Competitive adhesion studies have shown that MUB interacts with specific muco-oligosaccharides and that MUB binding has little to no host specificity regarding mucus components^15^. The second strategy of mucus adhesion is mediated by fimbrial adhesion of commensal bacteria. For example, *Escherichia coli*, a commensal bacterium residing in the human gut, has the potential to act as an opportunistic pathogen. *E. coli* strains use extracellular fimbriae, which have a two-domain organization: lectin at the most external N-terminal domain and pilin at the C-terminus connecting to the rest of the fimbria. The affinity and specificity of the adhesion by fimbrial proteins are governed by recognition of mucus glycan epitopes, which are age-, organ-, and species-specific^16^. However, for many bacterial pathogens, binding to mucus is a crucial step in their colonization. Flagella, composed of flagellin arranged in helical chains, are an important evolved strategy for mucus adhesion during infection by some pathogens, and they play a critical role in biofilm formation^17^. Enterotoxigenic *E. coli* (ETEC) strains are major causes of morbidity and mortality due to diarrheal illness in developing countries. ETEC-secreted pathovar-specific proteins (such as EtpA, a two-partner adhesin conserved within the ETEC pathovar) can interact with both the tips of ETEC flagella and mucus glycans to form molecular bridges promoting bacterial adhesion and intestinal colonization of pathogens^18^. Flagella are used as virulence factors by many enteropathogenic bacteria (e.g., *Listeria monocytogenes, Vibrio cholerae, E. coli*, and *Salmonella typhimurium*) to traverse the mucus barrier, resulting in infection. Flagella-driven motility propels pathogens towards the epithelium and accelerates disease progression^19^. Many human pathogens, including *C*. *difficile*, pathogenic *E. coli, Neisseria meningitidis*, and *Streptococcus pneumoniae*, also employ phase-variable flagella and fimbriae to evade the host immune system and promote host colonization, persistence, motility, and virulence^20^.

**Table 1:** Gut microbiome adhesion to mucin O-glycans.

Gut microbiome	Adhesin	Mucin epitopes	Adhesin PDB entry	Reference
Commensal bacteria				
*Bifidobacterium bifidum*	Extracellular transaldolase, extracellular sialidase	Type A antigen [Fucα1,2(GalNAcα1,3)Galβ	N/D	^119^
*Bifidobacterium longum* subsp.	Family 1 solute binding proteins (F1SBPs)	Mucin O-glycans	N/D	^120^
*E. coli* Nissle	Flagellum	Mucin O-glycans	N/D	^121^
*Lactobacillus*	Mucin-binding protein (MucBP), pili	N-acetylneuraminic acid (Neu5Ac)	4 MT5	^13^
*Ruminococcus gnavus*	Sialic acid-binding carbohydrate-binding module (CBM40) of intramolecular trans-sialidase (RgNanH)	α2,3- or α2,6-Sialyllactose	6RAB, 6RB7, 6RD1	^122^
Pathogens				
*Clostridium difficile*	FliC, FliD, toxin A (TcdA)	Galα1,3Galβ1,4GlcNAc	2F6E	^123^
*Campylobacter jejuni*	Carbohydrate-lectin, FlaA, MOMP	Fucα1, 2Gal1, 4GlcNAc	N/D	^124^
*E. coli* UPEC CFT073	F9 fimbriae	Gal β1,3 N-GalNAc in core-1 and -2 O-glycans	6AS8, 6ARO, 6ARN, 6ARM, 6AOW, 6AOY, 6AOX, 5 LNG, 5 LNE	^125^
Enteropathogenic *E. coli* (EPEC) E2348/69	H6 flagella	Mucin-type core2 O-glycan	N/D	^126^
Uropathogenic *E. coli*	PapG	GalNAcβ1,3 Galα1,4Galβ1,4Glc	1J8S, 1J8R	^127^
Enterotoxigenic *E. coli*, ETEC	F17-G flagella	GlcNAcβ1,3Gal	1O9Z, 1O9 W, 1O9 V, 1ZPL	^128^
Shiga toxin-producing *E. coli* (STEC)	F18 fimbrial subunit FedF	H antigens of type 1(Fucα1,2Galβ1,3GlcNAc)	4B4P, 4B4Q, 4B4R	^129^
Enterohemorrhagic *E. coli* (EHEC)	FimH	Mannose	1KIU, 1KLF	^130^
*Salmonella enterica* serotype	Fimbrial adhesin	α(1,2)fucose	N/D	^131^
*Listeria monocytogenes*	LPXTG-internalin proteins(MucBP), LmiA	N/D	2KT7	^132^
*Candida glabrata*	Lectin-like epithelial adhesin 1 (Epa1)A	T-antigen	4D3 W	^133^
	Epa6A	Lactose, T-antigen, N-acetyl-D-lactosamine, lacto-N-biose, α1,3-galactobiose,Galβ1,4GlcNAc	4COU, 4COW, 4COY, 4COZ, 4COV	
	Epa9A	Galβ1,4GlcNAc, lactose	4CP2, 4CP0	

### The gut microbiome feeds on mucin glycans

After adhesion to mucins, colonization by colonic bacteria is initiated, while the degradation of diverse and structurally complex mucin glycans depends on the cooperative action of sialidases, sulfatases, proteases, and glycoside hydrolases (GHs) encoded by the genomes of mucin-degrading bacteria (Fig. 1c). Mucin-degrading carbohydrate-active enzyme (CAZyme) families include sialidases (GH33), fucosidases (GH29, GH95), blood-group endo-β-1,4-galactosidases (GH98), mucin core GHs (GH101, GH129, GH84, GH85, and GH89), and sulfatases (GH20, GH2, GH42, unclassified)^21^ (Table 2). Carbohydrate-binding modules (CBMs) in CAZymes mediate their adherence to carbohydrate substrates in mucin polymers^22^.

**Table 2:** Gut microbiome enzymes involved in mucin degradation^134,135^.

Major phylum	Organism	Domains	PDB entry	Mucolytic enzyme
Bacteroidetes	*Alistipes finegoldii* DSM 17242	GH2, GH20,GH29		β-galactosidase (EC 3.2.1.23); β-1,6-N-acetylglucosaminidase (EC 3.2.1.-), β-6-SO3-N-acetylglucosaminidase (EC 3.2.1.-);α-1,3/1,4-L-fucosidase (EC 3.2.1.111)
	*Bacteroides caccae* ATCC 43185	GH2, GH20, GH29, GH33, GH35, GH84, GH89, GH95		β-galactosidase (EC 3.2.1.23); β-1,6-N-acetylglucosaminidase (EC 3.2.1.-); β-6-SO3-N-acetylglucosaminidase (EC 3.2.1.-); α-1,3/1,4-L-fucosidase (EC 3.2.1.111); sialidase or neuraminidase (EC 3.2.1.18); β-1,3-galactosidase (EC 3.2.1.-); [protein]-3-O-(GlcNAc)-L-Ser/Thr β-N-acetylglucosaminidase (EC 3.2.1.169); α-N-acetylglucosaminidase (EC 3.2.1.50); α-1,2-L-fucosidase (EC 3.2.1.63)
	*Bacteroides thetaiotaomicron* VPI-5482	GH2, GH20, GH29, GH33, GH35, GH42, GH84, GH89, GH95,	4BBW (GH33); GH29 (3EYP, 4OUE, 4OZO); GH84(2CHN, 2CHO, 2J47, 2J4G, 2JIW, 2 VVN, 2 VVS, 2 W4X, 2 W66, 2 W67, 2 WCA, 2 WZH, 2 WZI, 2X0H, 2XJ7, 2XM1, 2XM2, 4AIS,4AIU)	β-galactosidase (EC 3.2.1.23); β-1,6-N-acetylglucosaminidase (EC 3.2.1.-); β-6-SO3-N-acetylglucosaminidase (EC 3.2.1.-); α-1,3/1,4-L-fucosidase (EC 3.2.1.111); sialidase or neuraminidase (EC 3.2.1.18); β-1,3-galactosidase (EC 3.2.1.-); β-galactosidase (EC 3.2.1.23); [protein]-3-O-(GlcNAc)-L-Ser/Thr β-N-acetylglucosaminidase (EC 3.2.1.169); α-N-acetylglucosaminidase (EC 3.2.1.50); α-1,2-L-fucosidase (EC 3.2.1.63)
	*Bacteroides xylanisolvens* H207	GH2, GH20, GH29, GH33, GH35, GH42, GH89, GH95	—	β-galactosidase (EC 3.2.1.23); β-1,6-N-acetylglucosaminidase (EC 3.2.1.-); β-6-SO3-N-acetylglucosaminidase (EC 3.2.1.-); α-1,3/1,4-L-fucosidase (EC 3.2.1.111); sialidase or neuraminidase (EC 3.2.1.18); β-1,3-galactosidase (EC 3.2.1.-); β-galactosidase (EC 3.2.1.23); α-N-acetylglucosaminidase (EC 3.2.1.50); α-1,2-L-fucosidase (EC 3.2.1.63)
	*Odoribacter splanchnicus* DSM 20712	GH2, GH20, GH29, GH95,	—	β-galactosidase (EC 3.2.1.23); β-1,6-N-acetylglucosaminidase (EC 3.2.1.-); β-6-SO3-N-acetylglucosaminidase (EC 3.2.1.-); α-1,3/1,4-L-fucosidase (EC 3.2.1.111); α-1,2-L-fucosidase (EC 3.2.1.63)
	*Parabacteroides distasonis* ATCC 8503	GH2, GH20, GH29, GH33, GH95	—	β-galactosidase (EC 3.2.1.23); β-1,6-N-acetylglucosaminidase (EC 3.2.1.-); β-6-SO3-N-acetylglucosaminidase (EC 3.2.1.-); β-1,3-galactosidase (EC 3.2.1.-); α-1,2-L-fucosidase (EC 3.2.1.63)
	*Prevotella denticola* F0289	GH2, GH20, GH29, GH33, GH84, GH85, GH95	—	β-galactosidase (EC 3.2.1.23); β-1,6-N-acetylglucosaminidase (EC 3.2.1.-); β-6-SO3-N-acetylglucosaminidase (EC 3.2.1.-); α-1,3/1,4-L-fucosidase (EC 3.2.1.111); sialidase or neuraminidase (EC 3.2.1.18); [protein]-3-O-(GlcNAc)-L-Ser/Thr β-N-acetylglucosaminidase (EC 3.2.1.169); endo-β-N-acetylglucosaminidase (EC 3.2.1.96); α-1,2-L-fucosidase (EC 3.2.1.63)
	*Bacteroides fragilis* 638 R	GH2, GH20,GH29, GH33, GH35, GH84, GH89, GH95	—	β-galactosidase (EC 3.2.1.23); β-1,6-N-acetylglucosaminidase (EC 3.2.1.-); β-6-SO3-N-acetylglucosaminidase (EC 3.2.1.-); α-1,3/1,4-L-fucosidase (EC 3.2.1.111); sialidase or neuraminidase (EC 3.2.1.18); β-1,3-galactosidase (EC 3.2.1.-); [protein]-3-O-(GlcNAc)-L-Ser/Thr β-N-acetylglucosaminidase (EC 3.2.1.169); α-N-acetylglucosaminidase (EC 3.2.1.50); α-1,2-L-fucosidase (EC 3.2.1.63)
Firmicutes	*Ruminococcus bromii* L2-63	—	—	
	*Ruminococcus torques*	GH2, GH95	—	β-galactosidase (EC 3.2.1.23); α-1,2-L-fucosidase (EC 3.2.1.63);
	*Ruminococcus sp*. SR1/5	GH2, GH29, GH42	—	β-galactosidase (EC 3.2.1.23); α-1,3/1,4-L-fucosidase (EC 3.2.1.111); β-galactosidase (EC 3.2.1.23)
	*Ruminococcus bicirculans* 80/3	GH2, GH95	—	β-galactosidase (EC 3.2.1.23); α-1,2-L-fucosidase (EC 3.2.1.63)
	*Streptococcus thermophilus*	GH2	—	β-galactosidase (EC 3.2.1.23)
	*Streptococcus sanguinis* CGMH010	—	—	
	*Streptococcus oralis*	—	—	sulfatase
	*Clostridium perfringens* ATCC 13124	GH2, GH20, GH29, GH33, GH84, GH85, GH89, GH95, GH101	4 L2E(GH33)	β-galactosidase (EC 3.2.1.23); β-1,6-N-acetylglucosaminidase (EC 3.2.1.-); β-6-SO3-N-acetylglucosaminidase (EC 3.2.1.-); α-1,3/1,4-L-fucosidase (EC 3.2.1.111); sialidase or neuraminidase (EC 3.2.1.18); [protein]-3-O-(GlcNAc)-L-Ser/Thr β-N-acetylglucosaminidase (EC 3.2.1.169); endo-β-N-acetylglucosaminidase (EC 3.2.1.96); α-N-acetylglucosaminidase (EC 3.2.1.50); α-1,2-L-fucosidase (EC 3.2.1.63); endo-α-N-acetylgalactosaminidase (EC 3.2.1.97)
	*Lactobacillus reuteri* 1B	GH2	—	β-galactosidase (EC 3.2.1.23)
	*Lactobacillus plantarum* 10CH	GH2, GH20, GH42	—	β-galactosidase (EC 3.2.1.23); β-1,6-N-acetylglucosaminidase (EC 3.2.1.-); β-6-SO3-N-acetylglucosaminidase (EC 3.2.1.-); β-galactosidase (EC 3.2.1.23);
	*Lactobacillus rhamnosus* 4B15	GH2, GH29, GH35		β-galactosidase (EC 3.2.1.23); α-1,3/1,4-L-fucosidase (EC 3.2.1.111); β-1,3-galactosidase (EC 3.2.1.-)
	*Blautia hansenii* DSM 20583	GH2, GH20, GH29, GH33, GH84, GH85, GH95, GH101	—	β-galactosidase (EC 3.2.1.23); β-1,6-N-acetylglucosaminidase (EC 3.2.1.-); β-6-SO3-N-acetylglucosaminidase (EC 3.2.1.-); α-1,3/1,4-L-fucosidase (EC 3.2.1.111); sialidase or neuraminidase (EC 3.2.1.18); [protein]-3-O-(GlcNAc)-L-Ser/Thr β-N-acetylglucosaminidase (EC 3.2.1.169); endo-β-N-acetylglucosaminidase (EC 3.2.1.96); α-1,2-L-fucosidase (EC 3.2.1.63); α-1,2-L-fucosidase (EC 3.2.1.63); endo-α-N-acetylgalactosaminidase (EC 3.2.1.97)
	*Butyrivibrio fibrisolvens* 16/4	GH2, GH35, GH42	—	β-galactosidase (EC 3.2.1.23); β-1,3-galactosidase (EC 3.2.1.-); β-galactosidase (EC 3.2.1.23)
	*Eubacterium rectale* ATCC 33656	GH2, GH42	—	β-galactosidase (EC 3.2.1.23); β-galactosidase (EC 3.2.1.23)
	*Eubacterium siraeum* 70/3	GH2, GH95	—	β-galactosidase (EC 3.2.1.23); α-1,2-L-fucosidase (EC 3.2.1.63)
	*Faecalibacterium prausnitzii* 942/30-2	GH2	—	β-galactosidase (EC 3.2.1.23)
	*Roseburia intestinalis* L1-82	GH2, GH20, GH29, GH35, GH42, GH85, GH95	—	β-galactosidase (EC 3.2.1.23); β-1,6-N-acetylglucosaminidase (EC 3.2.1.-); β-6-SO3-N-acetylglucosaminidase (EC 3.2.1.-); α-1,3/1,4-L-fucosidase (EC 3.2.1.111); β-1,3-galactosidase (EC 3.2.1.-); β-galactosidase (EC 3.2.1.23); endo-β-N-acetylglucosaminidase (EC 3.2.1.96); α-1,2-L-fucosidase (EC 3.2.1.63)
Proteobacteria	*Burkholderia ambifaria* AMMD	GH2, GH20, GH42	—	β-galactosidase (EC 3.2.1.23); β-1,6-N-acetylglucosaminidase (EC 3.2.1.-); β-6-SO3-N-acetylglucosaminidase (EC 3.2.1.-); β-galactosidase (EC 3.2.1.23)
	*Pseudomonas aeruginosa* 12-4-4(59)	—	—	sulfatase
	*Shigella flexneri* 113	GH2	—	β-galactosidase (EC 3.2.1.23)
	*Vibrio cholerae* 569B 395	1 W0	1W0P, 1 W0O (GH33)	sialidase or neuraminidase (EC 3.2.1.18)
	*Vibrio cholerae* 10432-62	GH2, GH20, GH33	—	β-galactosidase (EC 3.2.1.23); β-1,6-N-acetylglucosaminidase (EC 3.2.1.-); β-6-SO3-N-acetylglucosaminidase (EC 3.2.1.-); sialidase or neuraminidase (EC 3.2.1.18)
	*Proteus vulgaris* biosolid 26	GH33	—	sialidase or neuraminidase (EC 3.2.1.18)
	*Klebsiella oxytoca* AR_0028	GH2, GH42	—	β-galactosidase (EC 3.2.1.23); β-galactosidase (EC 3.2.1.23)
	*Enterobacter cloacae* 109	GH2, GH20	—	β-galactosidase (EC 3.2.1.23); β-1,6-N-acetylglucosaminidase (EC 3.2.1.-); β-6-SO3-N-acetylglucosaminidase (EC 3.2.1.-)
	*Desulfovibrio desulfuricans* ATCC 27774	—	—	
	*Escherichia coli HS*	GH2	—	β-galactosidase (EC 3.2.1.23)
	*Escherichia coli*	GH2	—	β-galactosidase (EC 3.2.1.23)
Actinobacteria	*Bifidobacterium angulatum* DSM 20098 = JCM 7096	GH2, GH42	—	β-galactosidase (EC 3.2.1.23); β-galactosidase (EC 3.2.1.23)
	*Bifidobacterium longum* subsp. infantis ATCC 15697	GH2, GH20, GH29, GH35, GH42, GH95	3 MO4 (GH29)	β-galactosidase (EC 3.2.1.23); -1,6-N-acetylglucosaminidase (EC 3.2.1.-); β-6-SO3-N-acetylglucosaminidase (EC 3.2.1.-); α-1,3/1,4-L-fucosidase (EC 3.2.1.111); β-1,3-galactosidase (EC 3.2.1.-); β-galactosidase (EC 3.2.1.23); α-1,2-L-fucosidase (EC 3.2.1.63)
	*Bifidobacterium longum subsp. longum* JDM301	GH2, GH20, GH29, GH35, GH42, GH85, GH95	—	β-galactosidase (EC 3.2.1.23); -1,6-N-acetylglucosaminidase (EC 3.2.1.-); β-6-SO3-N-acetylglucosaminidase (EC 3.2.1.-); α-1,3/1,4-L-fucosidase (EC 3.2.1.111); β-1,3-galactosidase (EC 3.2.1.-); β-galactosidase (EC 3.2.1.23); endo-β-N-acetylglucosaminidase (EC 3.2.1.96); α-1,2-L-fucosidase (EC 3.2.1.63)
	*Bifidobacterium bifidum JCM 1254*	GH2, GH20, GH29, GH33, GH42, GH89, GH95	2EAB, 2EAC, 2EAD, 2EAE(GH95)	β-galactosidase (EC 3.2.1.23); β-1,6-N-acetylglucosaminidase (EC 3.2.1.-); β-6-SO3-N-acetylglucosaminidase (EC 3.2.1.-); α-1,3/1,4-L-fucosidase (EC 3.2.1.111); sialidase or neuraminidase (EC 3.2.1.18); β-galactosidase (EC 3.2.1.23); α-N-acetylglucosaminidase (EC 3.2.1.50); α-N-acetylgalactosaminidase (EC 3.2.1.49)
	*Bifidobacterium adolescents* ATCC 15703	GH2, GH35, GH42	—	β-galactosidase (EC 3.2.1.23); β-1,3-galactosidase (EC 3.2.1.-); β-galactosidase (EC 3.2.1.23)
	*Bifidobacterium catenulatum* DSM 16992 = JCM 1194 = LMG 11043	GH2, GH42	—	β-galactosidase (EC 3.2.1.23); β-galactosidase (EC 3.2.1.23)
	*Bifidobacterium breve* UCC2003	GH2, GH20, GH33, GH35, GH42, GH95, GH129	—	β-galactosidase (EC 3.2.1.23); β-1,6-N-acetylglucosaminidase (EC 3.2.1.-); β-6-SO3-N-acetylglucosaminidase (EC 3.2.1.-); sialidase or neuraminidase (EC 3.2.1.18); β-1,3-galactosidase (EC 3.2.1.-); β-galactosidase (EC 3.2.1.23); α-1,2-L-fucosidase (EC 3.2.1.63); α-N-acetylgalactosaminidase (EC 3.2.1.49)
	*Rothia mucilaginosa* DY-18	—	—	—
	*Bifidobacterium animalis* BL3	GH2, GH42		β-galactosidase (EC 3.2.1.23); β-galactosidase (EC 3.2.1.23)
Verrucomicrobia	*Akkermansia muciniphila* ATCC BAA-835	GH2, GH20, GH29, GH33, GH35, GH84, GH89, GH95		β-galactosidase (EC 3.2.1.23); β-1,6-N-acetylglucosaminidase (EC 3.2.1.-); β-6-SO3-N-acetylglucosaminidase (EC 3.2.1.-); α-1,3/1,4-L-fucosidase (EC 3.2.1.111); sialidase or neuraminidase (EC 3.2.1.18); β-1,3-galactosidase (EC 3.2.1.-); [protein]-3-O-(GlcNAc)-L-Ser/Thr β-N-acetylglucosaminidase (EC 3.2.1.169);α-N-acetylglucosaminidase (EC 3.2.1.50); -1,2-L-fucosidase (EC 3.2.1.63)
	*Akkermansia muciniphila* YL44	GH2, GH20, GH29, GH33, GH35, GH84, GH89, GH95	—	β-galactosidase (EC 3.2.1.23); β-1,6-N-acetylglucosaminidase (EC 3.2.1.-); β-6-SO3-N-acetylglucosaminidase (EC 3.2.1.-); α-1,3/1,4-L-fucosidase (EC 3.2.1.111); sialidase or neuraminidase (EC 3.2.1.18); β-1,3-galactosidase (EC 3.2.1.-); [protein]-3-O-(GlcNAc)-L-Ser/Thr β-N-acetylglucosaminidase (EC 3.2.1.169);α-N-acetylglucosaminidase (EC 3.2.1.50); -1,2-L-fucosidase (EC 3.2.1.63)
	*Akkermansia glycaniphila*	GH2, GH20, GH29, GH33, GH35, GH84, GH89, GH95	—	β-galactosidase (EC 3.2.1.23); β-1,6-N-acetylglucosaminidase (EC 3.2.1.-); β-6-SO3-N-acetylglucosaminidase (EC 3.2.1.-); α-1,3/1,4-L-fucosidase (EC 3.2.1.111); sialidase or neuraminidase (EC 3.2.1.18); β-1,3-galactosidase (EC 3.2.1.-); [protein]-3-O-(GlcNAc)-L-Ser/Thr β-N-acetylglucosaminidase (EC 3.2.1.169); α-N-acetylglucosaminidase (EC 3.2.1.50); α-1,2-L-fucosidase (EC 3.2.1.63)
	*Akkermansia muciniphila* AMDK-3	GH2, GH20, GH29, GH33, GH35, GH84, GH89, GH95	—	β-galactosidase (EC 3.2.1.23); β-1,6-N-acetylglucosaminidase (EC 3.2.1.-); β-6-SO3-N-acetylglucosaminidase (EC 3.2.1.-); α-1,3/1,4-L-fucosidase (EC 3.2.1.111); sialidase or neuraminidase (EC 3.2.1.18); β-1,3-galactosidase (EC 3.2.1.-); [protein]-3-O-(GlcNAc)-L-Ser/Thr β-N-acetylglucosaminidase (EC 3.2.1.169); α-N-acetylglucosaminidase (EC 3.2.1.50); α-1,2-L-fucosidase (EC 3.2.1.63)

The adult gut microbiome consists of hundreds to thousands of different species of bacteria, with two predominant bacterial phyla: gram-positive Firmicutes and gram-negative Bacteroidetes^23^. *Bacteroides* spp. are prominent members of this microbial ecosystem and widely studied commensal bacteria^24^. They degrade a vast range of dietary and endogenous glycans by utilizing a complex transenvelope machinery known as starch utilization system (Sus)-like systems, which are encoded by coregulated clusters of genes known as polysaccharide utilization loci (PULs)^25^. *Bacteroides* spp., in particular *B. thetaiotaomicron* containing PULs, encode highly specific CAZymes and degrade a wide range of glycan substrates, thereby stratifying the niche space with different orders of substrate preferences, which is why they are sometimes referred to as “generalists”^26^. *Akkermansia muciniphila* can hydrolyze up to 85% of mucin structures using different enzyme combinations^27^, strengthen intestinal epithelial integrity, and fortify damaged gut barriers^28^. Interestingly, the abundances of *A. muciniphila* in both fecal samples and mucosal biopsies of UC patients are markedly reduced^29^. Butyrate, an SCFA produced by commensal bacteria, is the main energy source of colonocytes and exerts various beneficial effects, such as enhancement of intestinal barrier function. The production of butyrate using complex mucin glycans as a substrate is generally restricted to some *Clostridium* clusters (IV and XIVa) from the Firmicutes phylum. In addition, the butyrogenic effect of *A. muciniphila*^30^ is related to its cross-feeding with mucus-degrading *Clostridium* clusters (IV and XIVa).

Notably, continual glycan degradation mediated by bacterial glycosidases may cause the disappearance of host-specific glycan epitopes and degradation of the protein backbone (Table 1). Dietary fiber-deprived intestinal microbiota have been shown to actively forage on the mucus layer, leading to dysfunction of the intestinal barrier and increased host susceptibility to pathogens and inflammation^10^ (Fig. 1c). It was reported that pathogenic *Proteobacteria* and *Firmicutes* species, including *Salmonella enterica* serovar *Typhimurium, E. coli*, and *C. difficile*, can benefit from cross-feeding through consumption of sialic acids from mucin molecules released by *B. thetaiotaomicron*. The expansion of pathogens during colitis is directly dependent on sialic acid released from host glycans catalyzed by sialidases^31^. Oral administration of a sialidase inhibitor and low levels of intestinal α2,3-linked sialic acid decreased *E. coli* outgrowth and colitis severity in mice^32^. The cleavage site of the zinc metalloprotease zmpB from *C. perfringens* was established to be next to the mucus glycoprotein backbone (Ser and/or Thr residues), with optimal splicing of GlcNAcβ1–3 (Neu5Acα2–6), GalNAcα1, or GalNAcα1 (α2,6-sialylated core 1 or core-3 O-glycan)^33^.

### The gut microbiome modulates mucus layer dynamics

Mucin production was reported to be induced by the gut microbiome. SCFAs such as acetate, propionate, and butyrate, the fermentation products of commensal bacteria, enhance the synthesis of mucin and stimulated mucin secretion in mice^34^. Moreover, the stimulating effect of butyrate on *Muc2* expression is mediated via AP-1 at the *Muc2* promoter^35^. Lactic acid-based probiotics, containing *Lactobacilli* and *Bifidobacteria*, increase mucin production in human intestinal epithelial cells and block enteropathogenic *E. coli* invasion and adherence in vitro^36^. Bifidobacterium species colonizing the intestinal mucus barrier modulate mucus production and expulsion by increasing the expression of GC markers such as Krüppel-like factor 4 (KLF4), trefoil factor 3 (TFF3), resistin-like molecule-beta (Relm-β), and Muc 2^37^. A randomized, placebo-controlled trial tested the efficacy and safety of a highly concentrated mixture of probiotic bacterial strains (VSL#3) in active UC and its role in the maintenance of UC remission^38^ and demonstrated that the protective effect of VSL#3 was related to enhanced colonic mucin expression and secretion in vivo and in vitro^39^. Several bacterial Toll-like receptor (TLR) ligands or effectors (e.g., lipopolysaccharide (LPS), flagellin, probiotic agents, commensal bacteria, and bacterial fermentation products) have been shown to trigger *Muc2* expression in colonic sGCs^5,40^. In addition, Muc2 production can also be enhanced by several stimuli, including T-helper type 1 (Th1)- and Th2 cell-mediated cytokines, acute phase responses (colonic ischemia), and viral infection^41,42^.

The gut microbiome is also involved in the modulation of mucus secretion by GCs. Non-O-glycosylated mucins with molecular weights of approximately 500 kDa are synthesized in the endoplasmic reticulum of GCs and dimerized via disulfide bonds between the cystine knot (CK) domains. Mucin dimers transported to the Golgi apparatus are subjected to O-glycosylation and then multimerization by disulfide bonds at N-terminal von Willebrand factor type D3 (vWF D3) domains. The resulting polymers reach molecular weights of up to 2.5 million Da^43^. Mucin multimers of 10–50 MDa [extended rods 1–10 μm in length] are then packaged in an ordered state within secretory vesicles (<1 μm) in the presence of low pH and high calcium^44^ (Fig. 1a). Upon secretion, the densely packed mucins can expand >1,000-fold, resulting in the formation of enormous net-like polymeric sheets^45^. Secretion of mucin can occur in at least two ways: regulated vesicle secretion and compound exocytosis. During regulated vesicle secretion (also called regulated exocytosis), the membrane of a secretory vesicle fuses with the plasma membrane by mediating the actions of typical vesicle exocytosis components such as syntaxins, mammalian uncoordinated-18 (Munc-18), vesicle-associated membrane proteins (VAMP), and synaptosome-associated proteins (SNAP), and this is a tightly controlled process most often triggered by calcium^46^. In compound exocytosis, storage vesicles rapidly fuse with the GC membrane after fusion with each other and empty all thecal contents^47^. The inner mucus layer is continuously renewed by mucin secretion of the surface GCs, and renewal of the inner mucus layer is estimated to occur every 1–2 h in live murine distal colonic tissue^48^. In general, spontaneous mucus production occurs at a rate of 240 μm/h in humans and 100 μm/h in the mouse colon; thus, the colonic mucus is continuously renewed at an average of 5–10 L per day^49^. Recently, sGCs have been shown to endocytose bacteria-derived TLR agonists such as LPS, lipid A, and flagellin but not lipotechoic acid, bacterial DNA, muramyl dipeptide, or γ-D-glutamylmeso-diaminopimelic acid and activate TLR- and MyD88-dependent NOD-like receptor family pyrin domain containing 6 (NLRP6) signaling to facilitate the exocytosis of mucin and flush bacteria away from crypt openings ex vivo^5^.

Stratification of the mucus layer has been shown to be indirectly influenced by the gut microbiome (Fig. 1a). An increase in pH and removal of N-terminally bound single calcium ions are necessary for the conversion of the inner firm mucus layer to the outer loose mucus layer, the so-called mucus layer stratification^50^. In general, colonocytes are mainly dependent on adenosine triphosphate produced by the β-oxidation of butyrate, a metabolite of the gut microbiome, which is accompanied by the generation of CO_2_ that can be converted by carbonic anhydrase into HCO_3_^−^^51^; this is the ideal physiological solution for precipitating calcium and raising the pH at the epithelial surface^52^. The absence of HCO_3_^−^ at the intestinal epithelial surface or inhibition of HCO_3_^−^ transepithelial transport decreases the amounts and rates of stimulated mucus release in vitro and in vivo^53^. For instance, facultative anaerobic bacteria such as pathogenic *E. coli* and *Salmonella* expand and invade the surface epithelium, thereby subverting colonocyte metabolism from β-oxidation of SCFAs to anaerobic glycolysis to promote their own luminal growth in competition against the gut microbiota by increasing the luminal bioavailability of oxygen (O_2_), lactate, and additional electron acceptors, including tetrathionate (S_4_O_6_^2−^) and nitrate (NO_3_^−^)^51,54^. The resultant decrease in HCO_3_^−^ in the lumen creates a high-H^+^ environment, enhancing the Ca^2+^-binding of mucin polymers and making them more adhesive to each other in condensed mucin granules^55^. As a result, the structure of mucus layers is impaired, and host susceptibility to pathogens and even UC incidence increases; therefore, UC was postulated to be an energy-deficient disease resulting from a failure to utilize butyrate^56^.

## The mucus barrier regulates bacterial colonization

### The mucus layer creates a habitat for commensal bacterial colonization

**Fig. 2:**
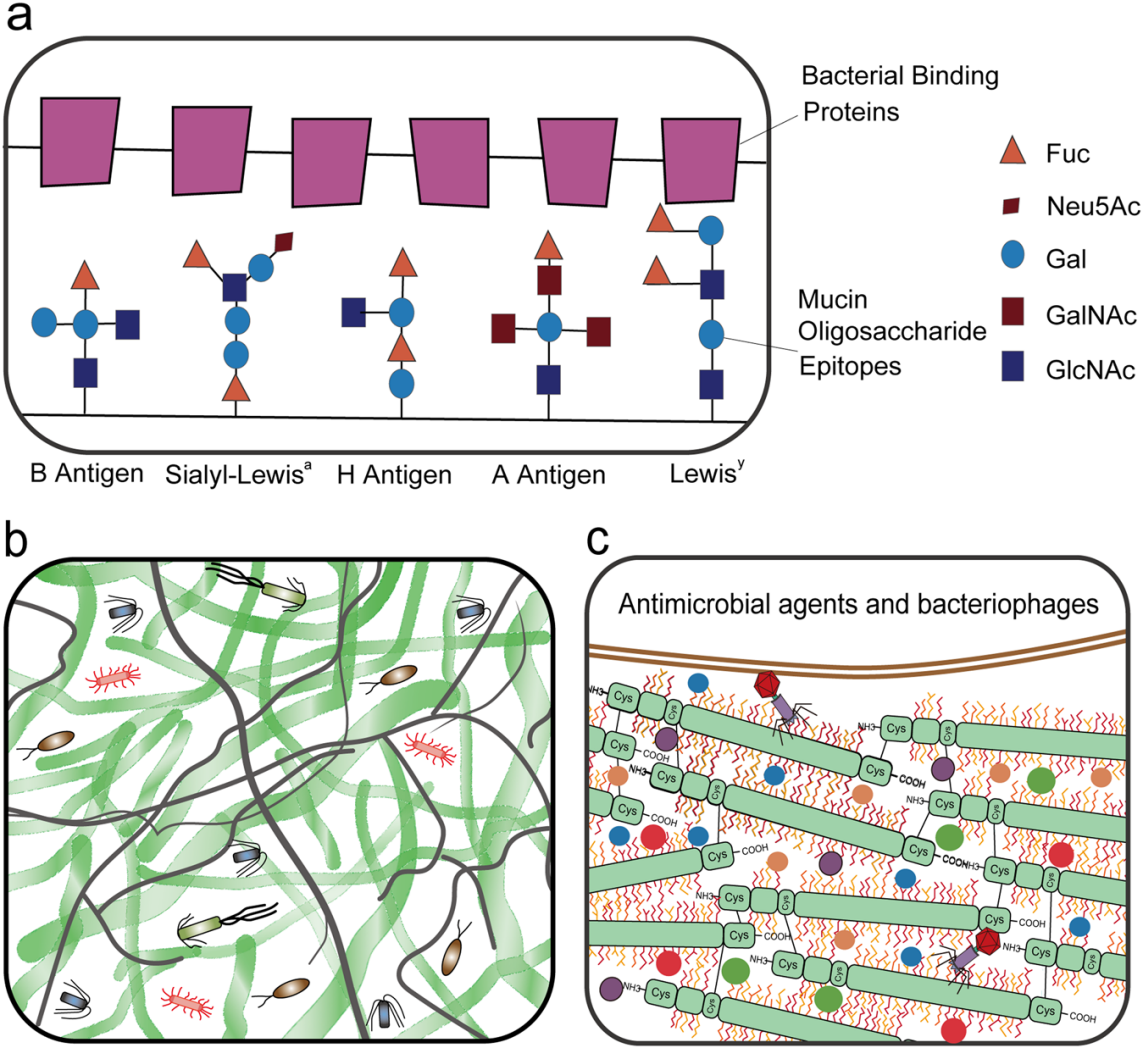
The mucus barrier functions to modulate bacterial colonization. **a** The mucus barrier forms a fundamental niche for gut microbiome colonization, where the major O-glycan epitopes are sialic acid, fucose, N-acetylneuraminic acid (Neu5Ac), type A antigen [GalNAcα1,3(Fucα1,2)Galβ], and type 1 H antigens [Fucα1,2Galβ1,3(GlcNAc)]. **b** The mucus barrier dictates the spatial organization of microbes, forming a steric and orderly microorganism network to inhibit pathogen colonization. **c** The mucus barrier is also a scaffold containing antimicrobial agents [including RELM-β (purple solid circle), ZG16 (blue solid circle), Ang4 (red solid circle), Lypd8 (green solid circle), sIgA (orange solid circle), and bacteriophages] protecting epithelial cells against microbes.

Hosts have evolved multiple strategies to maintain homeostasis of the intestinal microbiota (Fig. 2a). The best strategy is a highly adaptable protective mucus barrier exhibiting a heterogeneous spatial structure that establishes a habitat for commensal bacteria (Fig. 2b). The mucus barrier is a natural defense at the interface between host tissue and the luminal microbial community. Muc2 is the basic component of mucus that is continuously secreted and replenished by GCs in the large intestine. In the endoplasmic reticulum, the amino-terminal vWF and carboxy-terminal cystine knot (CK) domains of Muc2 mediate disulfide crosslinking of mucins to build a much larger mucin fishnet comprising thousands of monomers^57,58^. Muc2 consists of multiple domains, including the PTS [proline (Pro), threonine (Thr), and serine (Ser)] domain, a hallmark of the mucin family that is composed of a variable number of tandem repeats (VNTRs) that allow for heavy O-glycosylation with great heterogeneity in the Golgi apparatus and a stretched, brush-like arrangement of mucin. Neutral or negatively charged sugars, including N-acetylgalactosamine (GalNAc), sulfated acetyl-D-glucosamine (GlcNAc), D-galactose (Gal), sulfated Gal, sialic acid (Neu5Ac), and fucose, are attached to the PTS domains under catalysis by glycosyltransferases in the Golgi apparatus. Ultimately, these glycans account for up to 80% of the total mucin mass^21^. Importantly, the vast repertoire of O-glycosylated epitopes derived from the peripheral terminus of mucins (such as sialic acid and fucose) creates a habitat for unique bacterial ecosystems that thrive in proximity to host tissue^59,60^. Species of Bacteroides, the most abundant genus of the human gut microbiome, have a unique class of polysaccharide-utilizing loci that are referred to as commensal colonization factors (CCFs). *Bacteroides fragilis* can penetrate the colonic mucus and reside deep within crypt channels, whereas strains with CCF mutations are defective in crypt invasion^61^. It is known that reestablishment and resilience are fundamental characteristics of the gut microbial community^61,62^. The recolonization of gut *B. fragilis* following microbiome disruption caused by *Citrobacter rodentium* infection or antibiotic treatment is also dependent on CCFs^61,63^. Sulfatase (BF3086) and glycosyl hydrolase (BF3134) were annotated as mucosal colonization factors in *B. fragilis*. BF3086 is also important for *B. fragilis* to metabolize host mucus O-glycans^64^. During colonic mucus colonization, *B. fragilis* upregulates the expression of a set of candidate colonization factors, including BF3086 and BF3134, while in-frame deletions of these factors reduce its colonization abilities, which are fully or partially recovered by transcomplementation of BF3134 or BF3086^64^.

The inhibition of symbiotic bacterial colonization by pathogens is mediated by degradation of mucosal glycosylation and includes decreasing fucosylation and increasing the release of sialic acid, which promotes the outgrowth and colonization of pathogenic *E. coli*^32^. LPS induces an increase in the expression of microbial virulence genes, such as RtxA (K10953) and hemolysin III (K11068), which enhance intestinal colonization of pathogenic microbes in fucosyltransferase 2 (Fut2)-deficient mice^65^. Enterohemorrhagic *E. coli* (EHEC) encodes a two-component sensing system (FusKR) consisting of a histidine sensor kinase (FusK) and response regulator (FusR). During colonization, EHEC cleaves fucose from mucin, thereby activating the FusKR signaling cascade and increasing the expression of virulence genes^66^. It was observed that *S. typhimurium* had significantly increased expression of genes (*nan, fuc*, and *pdu*) that utilize host mucin monosaccharides such as sialic acid, fucose, and propanediol, the catabolite of fucose, in gnotobiotic mice colonized with sialidase-expressing *B. thetaiotaomicron*^67^. Furthermore, antibiotic-treated conventional mice exhibited a transient surge in free sialic acid liberated by the resident microbiota from host mucus, promoting the expansion of *Salmonella* and *C. difficile* expressing sialic acid catabolic signaling^67^. As a result, it was concluded that antibiotic-associated pathogens such as *S. typhimurium* and *C*. *difficile* catabolize fucose and sialic acid liberated by the resident microbiota from mucin glycans in a resident microbiota-dependent manner^67^. Pathogens have also evolved a range of mucin-hydrolyzing enzymes called mucinases (glycosidases, proteases, and sulfatases) to degrade mucin complexes due to the mucus net-like nature. Notably, some commensal bacteria also produce mucinases, but their expression levels are much lower (Fig. 1c). Compared to pathogenic *E. coli*, commensal *E. coli* strains generate a lower amount of YghJ^68^, a lipoprotein with a zinc metalloprotease domain that is involved in mucin degradation as well as proinflammatory responses.

The colonization of commensals at the mucus layer also renders host resistance to pathogen colonization. CCFs mediate the production of a polysaccharide capsule around *B. fragilis*, thereby initiating an IL-36γ response in mucosal macrophages of the gut to prevent colonization and infection by *Klebsiella pneumoniae*, which is a multidrug-resistant pathogen with high lethality^69^. Pathogens can be directly killed or inhibited by commensals that produce several antibacterial compounds. For example, bacteriocins produced by commensal *E. coli* inhibit EHEC^70^, microbicides secreted by *Enterobacteriaceae* mediate interspecies competition in the inflamed gut^71^, the bacteriocin thuricin produced by *Bacillus thuringiensis* inhibits the proliferation of *C. difficile* and *L. monocytogenes*^72^, and lantibiotics produced by lactic acid bacteria are used to target pathogens^73^. In addition, mucin was found to affect microbial behavior. For instance, gram-negative pathogens *V. cholerae*^74^ and *S. Typhimurium*^75^ as well as commensals from the *Bacteroides* genus^76^ were reported to exert bactericidal effects mediated by the Type VI secretory system (T6SS) (Fig. 1c). It was recently revealed that mucin-associated glycans activate RetS, the sensor kinase of *Pseudomonas aeruginosa*, thereby inhibiting T6SS-dependent bacterial killing action^77,78^.

### Epithelial surface pH modulates the gut microbiota composition

There are two key transport systems for HCO_3_^−^ extrusion into the colonic lumen: Cl^−^/HCO_3_^−^ and SCFA/HCO_3_^−^ exchangers^79^ (Fig. 1b). Several lines of evidence indicate that SCFA/HCO_3_^−^ exchangers mediate ionized SCFA entry into colonocytes concomitant with an increase in luminal pH and a decrease in oxygen tension in both human and rodent colons^80^, which are vital for the stratification of the secreted mucin complex and colonization of obligate anaerobes, respectively. Treatment with live *Bifidobacterium* and its culture supernatants stimulated the expression of Slc26a3, a Cl^−^/HCO_3_^−^ exchanger^81^. Inflammation in the mid-distal^82^ or distal colon^83^ in *Slc26a3*-deficient mice was related to the loss of mucus secretion resulting from a remarkably low surface pH microclimate^83^, a more aggressive microbiota^82^ and/or reduced microbiome diversity^83^. A luminal microenvironment with higher oxygen and lower pH could change the gut microbiota composition and drive an uncontrolled luminal expansion of *E. coli* and *Salmonella*^84^.

### Mucus viscosity determines the spatial organization of the gut microbiota

The intestinal microflora is not evenly mixed but is spatially organized (Fig. 2c). Some mechanisms for the spatial organization of gut bacteria have been elucidated. Mucus is mainly composed of water (95% w/w), mucins (0.2–5.0% w/v), globular proteins (0.5% w/v), salts (0.5–1.0% w/w), lipids (1–2% w/w), DNA, cells, and cellular debris that form a dense, viscoelastic layer over epithelial cells^85^. There is a longitudinal (proximal to distal colon) viscosity gradient that increases progressively towards the distal colon in murine models, which restricts bacterial motility and confers spatial organization of bacterial populations. As a result, bacteria are selectively separated from the mucosa in the proximal colon and completely separated in the mid-distal colon^86^. Of note, uncovered cecum epithelium tips are a hotspot for *S. typhimurium* infection in mice due to the lack of a continuous mucus layer^19^. In the proximal murine colon, select bacterial populations intimately contact the mucosa and enter the crypts, thereby concentrating and forming a 20–240-μm thick film flanking the mucosa. The existence of vertical (surface to lumen) viscosity gradients within the colonic mucus layer was further demonstrated by low mucus viscosity at the crypt base and high viscosity at sites adjacent to the columnar epithelium or close to the intestinal lumen. A viscosity-dependent spatial distribution of bacteria in the murine colon revealed that short rods and cocci moved best in low viscosity, while long curly bacteria preferred a moderately viscous environment, and all bacteria were immobilized by high viscosity^87^. The lower viscosity of mucus at the crypt base makes intestinal cells more vulnerable to invasion by potential pathogens. In general, mucins contain several crosslinking domains to form dimers and larger-order structures via disulfide bonds that may be broken by sulfate-reducing bacteria (SRB), particularly *Desulfovibrio desulfuricans*^88^. Many studies have described a high abundance of SRB detected in the mucosa of UC patients^89,90^. The resultant mucus barrier becomes less viscous and more permeable, allowing the gut microbiota in the gut lumen to interact with epithelial cells, thereby causing an aberrant immune response^91^. Recent studies have revealed the importance of site-specific gene expression for robust host-microbial symbiosis. *B. fragilis* near the epithelium upregulates the expression of genes involved in protein synthesis; moreover, compared to bacteria in the lumen, *B. fragilis* in mucus and tissue has high levels of sulfatase (BF3086) and glycosyl hydrolase (BF3134)^64^. Intestinal mechanics are a host spatial control measure capable of regulating the abundance and persistence of gut bacteria. A *V. cholerae* symbiont native to zebrafish that governs its spatial organization using swimming motility and chemotaxis displayed strong localization to the foregut region, an anatomical region comparable to the mammalian small intestine with close contact with the intestinal epithelium to counter intestinal flow. In contrast, motility-deficient mutants that are susceptible to host spatial control largely aggregated within the intestinal mucus and were confined to the lumen, whereas chemotaxis-deficient mutants were restricted to the lumen of the midgut, and two mutants were susceptible to intestinal expulsion. Wild-type *V. cholerae* actively escapes mucus through regular changes in swimming direction mediated by chemotactic signaling^92^.

There are some factors influencing the viscosity of the mucus layer, TFF3 and HCO_3_^−^. TFF3, as a component of mucus, is essential for protection of the gastrointestinal mucosa^93^. It is a small cysteine-rich acidic secreted protein that is covalently bound to the C-terminal domain of Muc2^94^. Mucus viscosity has been shown to increase after the introduction of TFF3 dimers (0.3% w/v) compared with no treatment^95^. *Tff3-*knockout mice are more susceptible to dextran sulfate sodium (DSS)-induced colitis^96,97^, while oral treatment with TFF3 protected against DSS-induced colitis in mice^93^.

There are two separate signaling pathways vital for normal mucus formation: Ca^2+^-mediated exocytosis of mucin granules of GCs and independent cAMP-mediated, cystic fibrosis transmembrane conductance regulator (CFTR)-dependent HCO_3_^−^ secretion, which helps discharge sulfated and sialylated glycosylated domains^85^ and stratifies exocytosed mucus^98^. Additionally, HCO_3_^−^ also participates in mucin expansion and hydration mechanisms by reducing Ca^2+^ cross-linking in mucins, thereby decreasing the viscosity^55^. CFTR is the secretory chloride/HCO_3_^−^ channel; its dysfunction causes acidification of the mucus layer (pH < 6.5) due to defective HCO_3_^−^ release, resulting in increased mucus viscoelasticity and the formation of a stationary mucus layer in cystic fibrosis^99^.

### The mucus barrier generates a protective shield

Colonic mucus is a key component of the colonic barrier, as it is located at the interface between luminal microflora and the colonic mucosa. The mucus barrier effectively partitions the enteric epithelium from the microbiota as the first line of defense and supports the growth of intestinal commensals as an energy source. The development of colitis in animals lacking a functional mucus layer closely reflects clinical and cellular features in patients with active UC. Penetration of the inner mucus layer in the distal colon by pathogens and/or commensals often found in mice with colitis is related to impaired mucus barrier structure and function caused by genetic deficiency in Muc2^100^, inactivation of glycosyltransferase-mediated O-glycosylation of Muc2^101,102^, deficiency of the NLRP6 inflammasome, or exposure to colitis-inducing chemicals^103^. Some pathogens such as enterohemorrhagic or enteropathogenic *E. coli* (EHEC or EPEC), *C. rodentium*, and *S. typhimurium* disrupt the protective mucus barrier, causing dysbiosis characterized by decreased abundances of *Firmicutes* and *Verrucomicrobia* and increased abundances of *Bacteroidetes* and facultative anaerobes^104^, which adhere to or invade host epithelial cells beneath the mucus layer. The vicious cycle of dysbiosis and colonic inflammation is characterized by destruction of the mucus barrier and persistent overstimulation of the immune system by the microflora^19^. Chronic or intermittent dietary fiber deficiency pushes the resident microbiota to rely more heavily on endogenous nutrients (host-secreted mucin glycoproteins), leading to erosion of the colonic mucus barrier and exacerbation of colitis triggered by the mucosal pathogen *C. rodentium*^10^.

### Antimicrobial agents fortify the mucus barrier

Importantly, the dense gel-forming structure of the mucus layer acts as a trap to stabilize numerous molecules, such as RELM-β and zymogen granule protein 16 (ZG16), angiogenin 4 (Ang4), Ly6/PLAUR domain containing 8 (Lypd8), and secretory immunoglobulin A (sIgA) (Fig. 2b). RELM-β exerts a microbicidal effect predominantly on gram-positive pathogens penetrating the mucus layer^104^. ZG16 prevents the adherence of bacteria to the epithelium by binding to the peptidoglycan of the bacterial cell wall^105^. Ang 4, another antimicrobial agent derived from GCs, is associated with *Trichuris muris* expulsion from the colonic epithelium of mice during inflammation^106^. *B. thetaiotaomicron* promotes Ang 4 expression, which inhibits the growth of some bacterial species, such as *L. monocytogenes* and *Enterococcus faecalis*^107^. Lypd 8, a highly glycosylated glycosylphosphatidylinositol-anchored protein selectively expressed in enterocytes, can bind to flagellated bacteria to inhibit bacterial invasion into the colonic epithelia when secreted into the lumen. Lypd8 strongly causes early-phase defense against *C. rodentium*, which can induce colitis by triggering attachment and effacement (A/E) lesions on colonic epithelia. Mechanistically, Lypd8 inhibits *C. rodentium* attachment to intestinal epithelial cells by binding to intimin, thereby protecting against enteric bacterial pathogens^108^. sIgA secreted as a dimer by colonocytes and integrated into the mucus layer exerts a critical function in trapping luminal bacteria to prevent unrestricted access of the microbiota to the epithelial surface^109^. The decreasing gradient of antimicrobial agents from the epithelial surface to the lumen is positively correlated with mucin concentration in the bilayered mucus matrix, which is why the intestinal mucus layers harbor significant antibacterial activity, whereas only low activity is detected in the luminal content. Because of the anti-inflammatory and antimicrobial nature of mucosal contents, the mucus layer generates a protective shield to prevent bacterial translocation and inappropriate immune stimulation of the epithelium^110^. However, when a functional mucus layer is absent, the gradient of antimicrobial agents is diminished, and the related defense system is eliminated from the intestine with fecal flow^23^.

### Bacteriophage attachment to mucus strengthens mucus defense

Under homeostatic conditions, mucus provides protection against dysbiosis by bacteriophage deployment (Fig. 2b). Bacteriophage, a resident member of the gut microbiome, interacts with mucin glycoproteins in the mucus barrier though immunoglobulin-like domains that are exposed on the capsid, triggering nonhost-derived immunity, which is considered part of the innate immune system^111^. Adherent invasive *E. coli* (AIEC) strain LF82 has type 1 pili mediating its binding to the host adhesion receptor carcinoembryonic antigen-related cell adhesion molecule 6 (CEACAM6), which is more strongly expressed in the ileal tissues of patients with Crohn’s disease (CD)^112^. A single day of oral treatment with a cocktail of bacteriophages was found to induce significantly decreased intestinal colonization by AIEC strain LF82 in CEABAC10 transgenic mice^113^. Moreover, this single dose of bacteriophage inhibited DSS-induced colitis symptoms over a two-week period in conventional mice colonized with LF82^114^. Bacteriophage intervention is planned to be evaluated in patients with IBD in the United States^113^. Data from UC mouse models have revealed that some bacteriophages that infect bacteria with pathogenic potential (pathobionts) are elevated during colitis^115^. Specifically, an increased abundance of bacteriophages predicted to infect *Streptococcus* sp. and *Alistipes* and *Clostridiales* phages predicted to infect *C. difficile* were observed during colitis^116^. This elevated abundance of specific phages could be postulated as a proxy for strain-level resolution of disease-causing bacteria during IBD^116^. It has been reported that intestinal microbiota-associated phages attach to mucins and protect underlying epithelial cells from invading bacteria^117^. Spatial organization of the mucus generates a gradient of phage replication with lysogeny at the top mucosal layer and lytic predation in the bacteria-sparse intermediary layers^117^. However, animals with bacteriophage expansion, such as *Caudovirales* phages, exhibit a significant exacerbation of intestinal colitis^118^. This inconsistency indicates a complex role of phages in IBD.

## Conclusion

Massive advances in the etiology of UC over the past few decades have improved our understanding of the importance of active communication between the gut microbiota and the mucus barrier. It is evident that disturbance of this interplay is a vital pathological factor for UC development. From the perspective of intricate interactions between the mucus barrier and the gut microbiome in the gut microenvironment, it is important to explore interventional approaches to control inflammation or promote FMT. Hence, exploring promising therapeutic agents from the viewpoint of ‘slimy’ partners is necessary to effectively treat UC.
